# Supplementary material for: Seminoma and Embryonal Carcinoma Footprints Identified by Analysis of Integrated Genome-Wide Epigenetic and Expression Profiles of Germ Cell Cancer Cell Lines
Source: PLoS One. 2014 Jun 2;9(6):e98330. doi: 10.1371/journal.pone.0098330 (PMC4041891; doi:10.1371/journal.pone.0098330)
Supplement: File S1 — ZIP file containing DMRforPairs output for significant regions. Please start from the html files. (ZIP) [file pone.0098330.s008.zip › figures/14465.pdf]

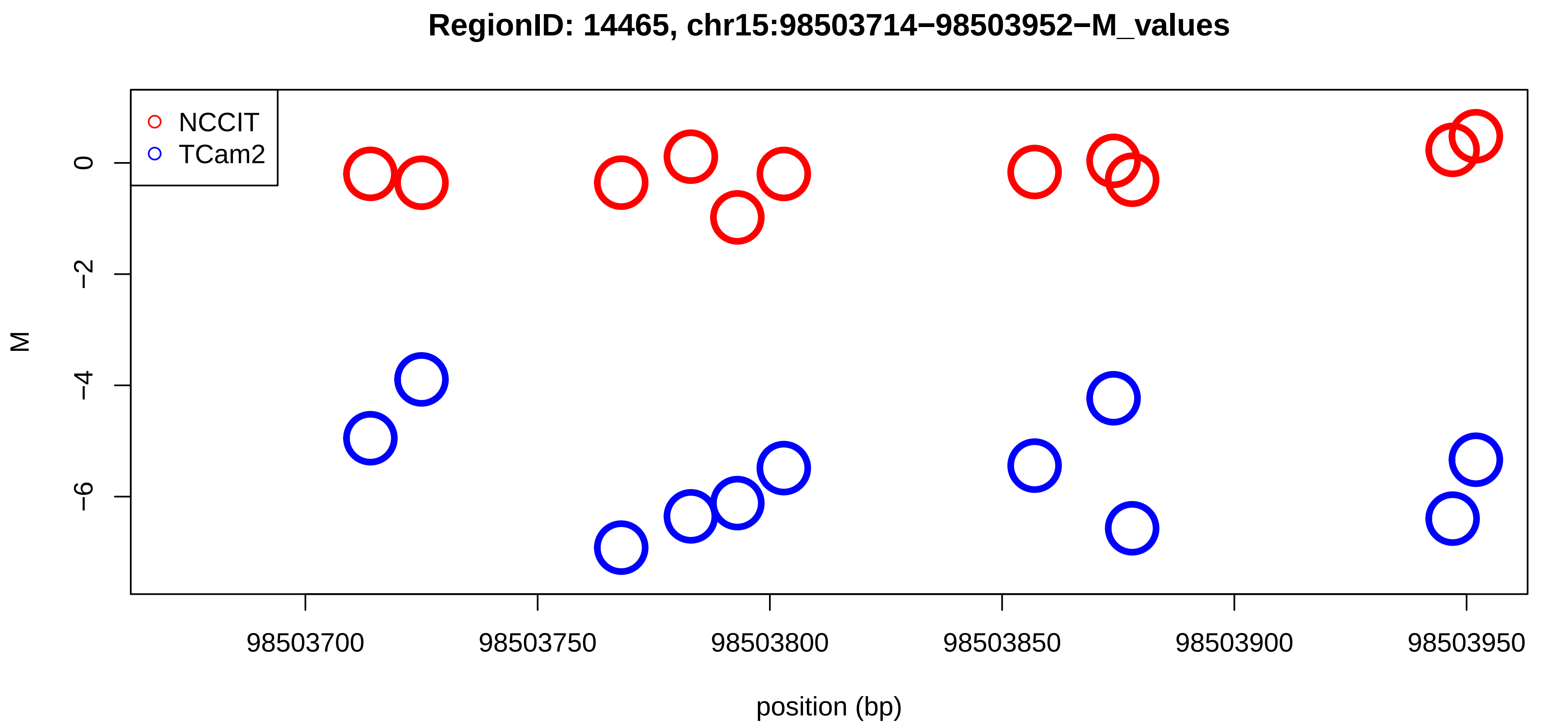

RegionID: 14465, chr15:98503714−98503952−M_values
NCCIT
TCam2
M
0
−2
−4
−6
98503700
98503750
98503800
98503850
98503900
98503950
position (bp)

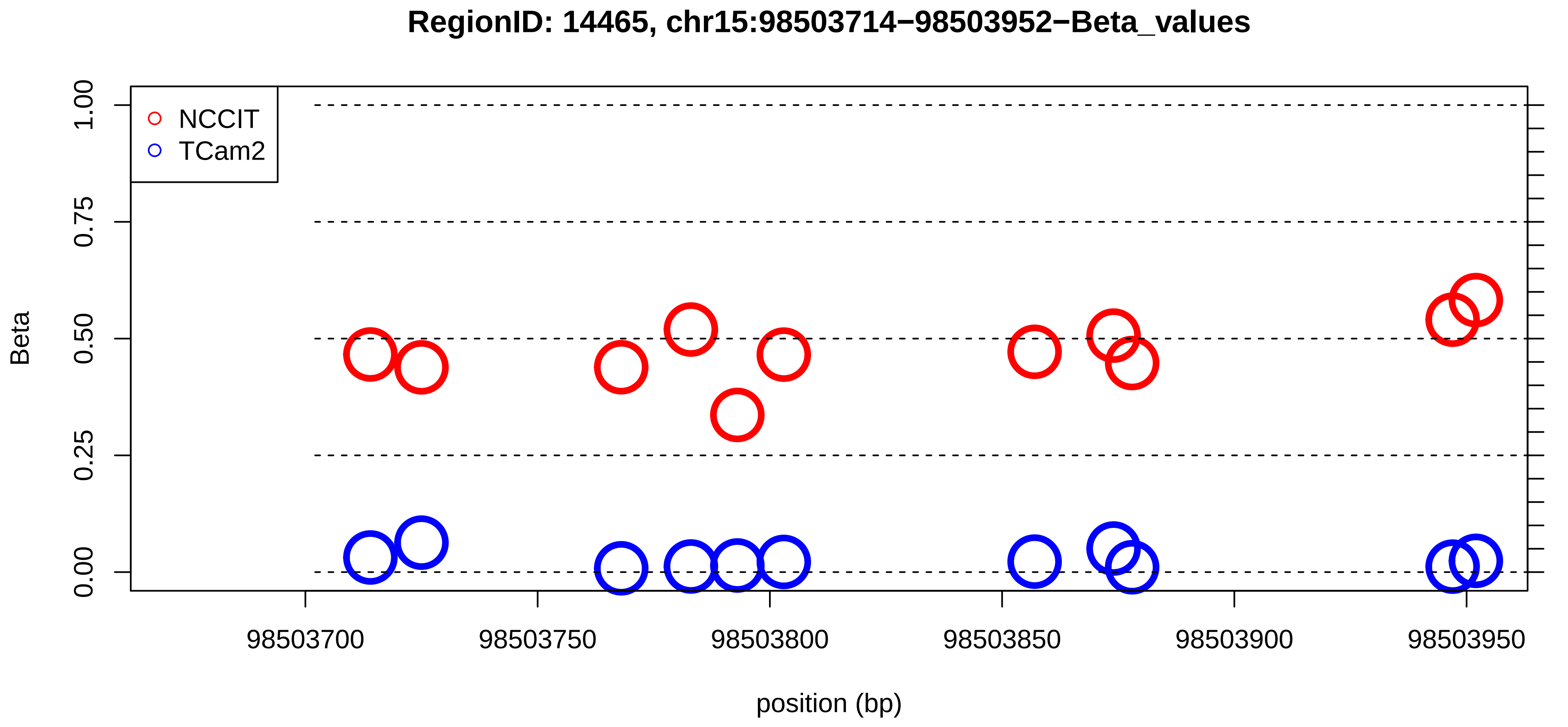

RegionID: 14465, chr15:98503714−98503952−Beta_values
NCCIT
TCam2
Beta
1.00
0.75
0.50
0.25
0.00
98503700
98503750
98503800
98503850
98503900
98503950
position (bp)

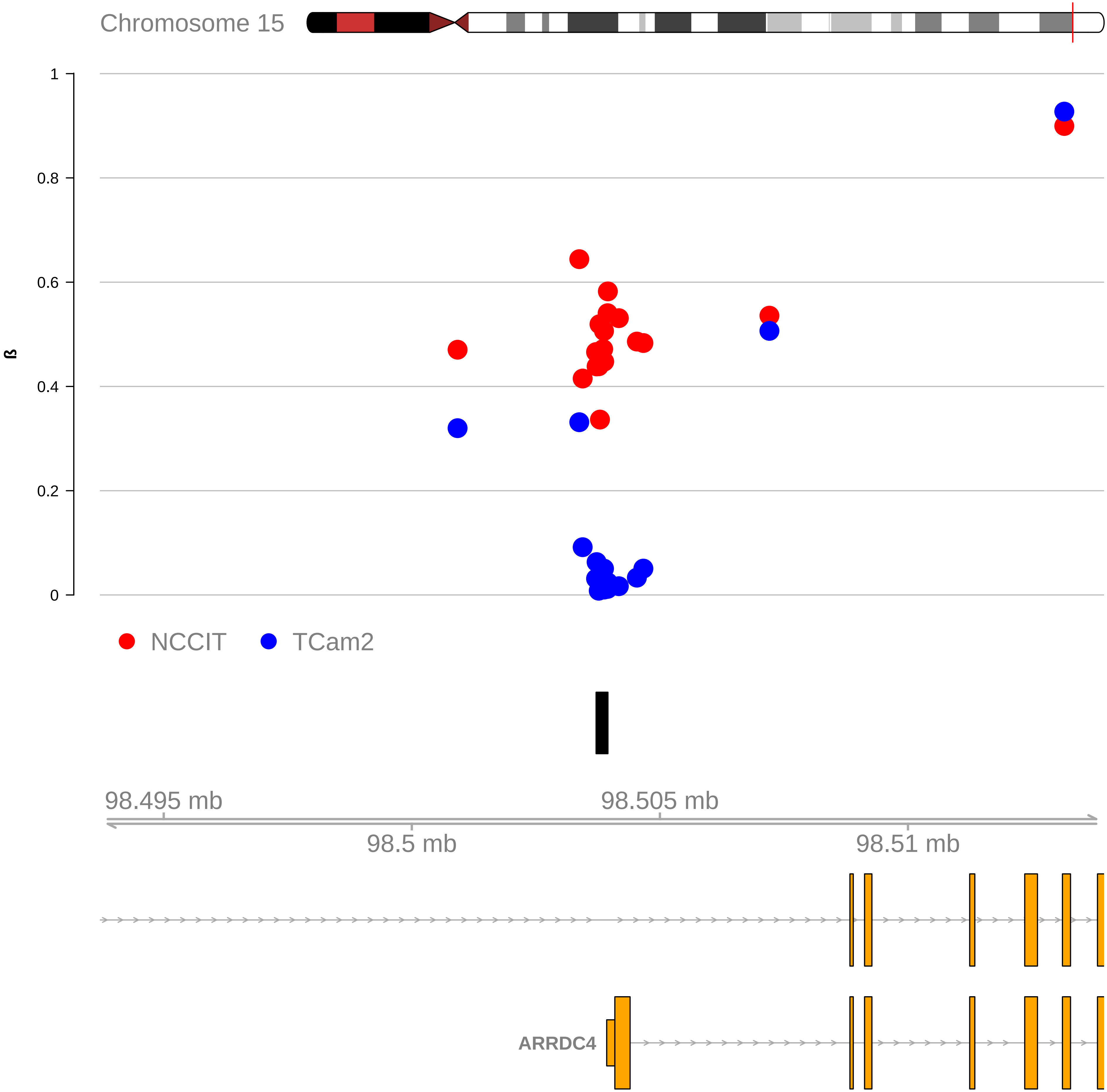

Chromosome 15
1
0.8
0.6
0.4
0.2
0
ß
NCCIT
TCam2
98.495 mb
98.505 mb
98.5 mb
98.51 mb
ARRDC4
